# Predictive value of FCGBP expression for treatment response and survival in rectal cancer patients undergoing chemoradiotherapy

**DOI:** 10.18632/aging.205791

**Published:** 2024-05-03

**Authors:** Yu-Ting Su, Chung-Hsing Chen, Jui-Wen Kang, Hsin-Yu Kuo, Ching-Chieh Yang, Yu-Feng Tian, Cheng-Fa Yeh, Chia-Lin Chou, Shang-Hung Chen

**Affiliations:** 1 Department of Oncology, National Cheng Kung University Hospital, College of Medicine, National Cheng Kung University, Tainan 70456, Taiwan; 2 National Institute of Cancer Research, National Health Research Institutes, Zhunan 35053, Taiwan; 3 Department of Internal Medicine, National Cheng Kung University Hospital, College of Medicine, National Cheng Kung University, Tainan 70456, Taiwan; 4 Institute of Clinical Medicine, College of Medicine, National Cheng Kung University, Tainan 70101, Taiwan; 5 Department of Radiation Oncology, Chi Mei Medical Center, Tainan 71004, Taiwan; 6 Department of Pharmacy, Chia-Nan University of Pharmacy and Science, Tainan 71710, Taiwan; 7 Division of Colon and Rectal Surgery, Department of Surgery, Chi Mei Medical Center, Tainan 71004, Taiwan; 8 Division of General Internal Medicine, Chi Mei Medical Center, Tainan 710, Taiwan; 9 Department of Environment Engineering and Science, Chia Nan University of Pharmacy and Science, Tainan 71710, Taiwan; 10 Department of Medical Technology, Chung Hwa University of Medical Technology, Tainan 71703, Taiwan; 11 National Institute of Cancer Research, National Health Research Institutes, Tainan 70456, Taiwan

**Keywords:** FCGBP, rectal cancer, chemoradiotherapy

## Abstract

Despite neoadjuvant chemoradiotherapy (CRT) being the established standard for treating advanced rectal cancer, clinical outcomes remain suboptimal, necessitating the identification of predictive biomarkers for improved treatment decisions. Previous studies have hinted at the oncogenic properties of the Fc fragment of IgG binding protein (FCGBP) in various cancers; however, its clinical significance in rectal cancer remains unclear. In this study, we first conducted an analysis of a public transcriptome comprising 46 rectal cancer patients. Focusing on cell adhesion during data mining, we identified *FCGBP* as the most upregulated gene associated with CRT resistance. Subsequently, we assessed FCGBP immunointensity using immunohistochemical staining on 343 rectal cancer tissue blocks. Elevated FCGBP immunointensity correlated with lymph node involvement before treatment (p = 0.001), tumor invasion, and lymph node involvement after treatment (both p < 0.001), vascular invasion (p = 0.001), perineural invasion (p = 0.041), and reduced tumor regression (p < 0.001). Univariate analysis revealed a significant association between high FCGBP immunoexpression and inferior disease-specific survival, local recurrence-free survival, and metastasis-free survival (all p ≤ 0.0002). Furthermore, high FCGBP immunoexpression independently emerged as an unfavorable prognostic factor for all three survival outcomes in the multivariate analysis (all p ≤ 0.025). Enriched pathway analysis substantiated the role of FCGBP in conferring resistance to radiation. In summary, our findings suggest that elevated FCGBP immunoexpression in rectal cancer significantly correlates with a poor response to CRT and diminished patient survival. FCGBP holds promise as a valuable prognostic biomarker for rectal cancer patients undergoing CRT.

## INTRODUCTION

Colorectal cancer (CRC) ranks as the third most prevalent cancer in males and the second most common cancer in females globally [1]. In 2020, it accounted for 1.9 million new cases, representing 10.7% of all new cancer diagnoses and earning its position as the third most frequent malignancy. Additionally, it stood as the third leading cause of cancer-related deaths in men and the fourth in women. Notably, radiotherapy holds significant therapeutic relevance for rectal cancers. Enhanced local disease control for individuals with locally advanced rectal cancer, characterized by stage T3–4 tumors or positive lymph nodes, can be achieved through the adoption of total mesorectal excision as the standard surgical approach, complemented by preoperative chemoradiotherapy (CRT) [2, 3]. Despite these advancements, the implementation of multimodal therapy has not translated into substantial improvements in overall survival [4]. Approximately 30% of patients with locally advanced rectal cancer experience relapse, often manifesting as distant metastases. To enhance therapeutic decision-making, the identification of prospective predictive biomarkers becomes imperative for optimizing treatment strategies and improving outcomes in this challenging patient population.

FCGBP, a substantial mucin-like glycoprotein secreted by goblet cells, was initially identified on the human intestinal mucosa as a distinctive binding site for the Fc region of IgG [5, 6]. Its structural composition shares similarities with other conventional mucin proteins, such as Mucin 2 (MUC2). These proteins are characterized by a high molecular weight (>200 kDa) and consist of numerous repeated domains, such as von Willebrand factor D domains, tandem repeat domains, and cysteine-rich units [6–8]. The distribution of FCGBP spans various tissues, including the colon, small intestine, gall bladder, cystic duct, choledochus, bronchus, submandibular gland, and uterine cervix, along with the corresponding secretory fluids [5]. While the precise function of FCGBP remains incompletely understood, it has been associated with innate immunity as an integral component of intestinal mucus—a primary defense mechanism in the gastrointestinal system [9–13]. Given the pivotal role of mucins in regulating bacterial adherence on mucosal surfaces, alterations in FCGBP may signify substantial structural modifications within the mucus.

Due to its pivotal roles in adhesion and immunologic functions, numerous studies have explored the evolving significance of FCGBP in the development of human malignancies. While diminished expression levels of FCGBP have been noted in gallbladder and prostate cancers [14, 15], contrasting observations reveal elevated FCGBP expression in glioma, hepatocellular carcinoma, and ovarian cancer [16–18]. The prognostic implications of FCGBP expression have been established in these malignancies, shedding light on its potential as a predictive biomarker. Notably, FCGBP expression has demonstrated associations with chemotherapy responses in patients with ovarian cancer [19], suggesting a modulatory role in the efficacy of contemporary anticancer modalities. The initial connections between FCGBP and CRC stem from its involvement in ulcerative colitis [20], a chronic inflammatory condition predisposing the individuals to CRC [21]. Subsequent investigations have unveiled that FCGBP expression significantly correlates with tumor metastasis and reduced overall survival in patients with CRC [22]. This finding underscores the potential of FCGBP as a predictive biomarker for assessing responses in patients with rectal cancer. Consequently, this study aims to establish correlations between FCGBP expression, CRT efficacy, and survival outcomes. Utilizing a combination of public databases and a large-scale Taiwanese rectal cancer cohort, our research seeks to provide valuable insights into the potential clinical implications of FCGBP in this context.

## RESULTS

### *FCGBP* emerges as the most significantly upregulated gene associated with cell adhesion in CRT-resistant rectal cancer

**Figure 1:**
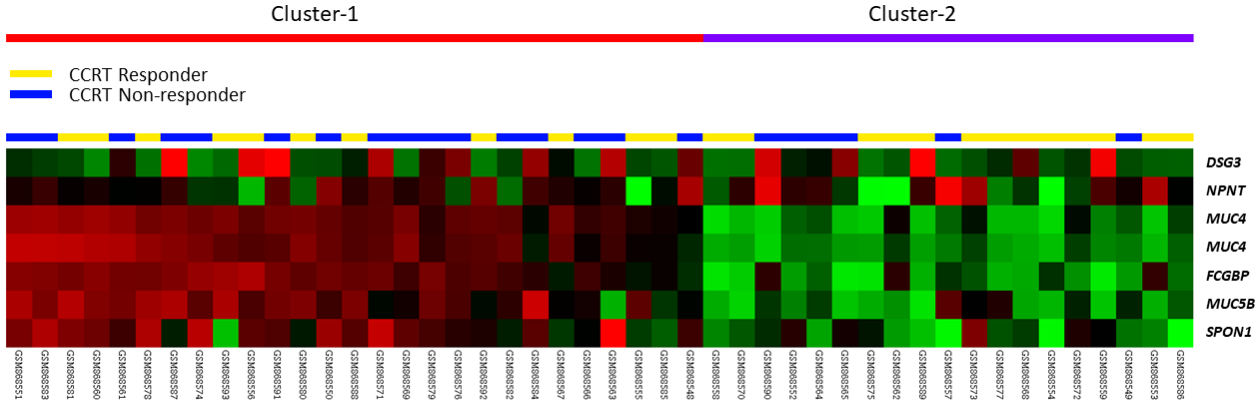
**Expression profiling of genes associated with cell adhesion (GO:0007155) and their relationship to chemoradiotherapy response.** We identified *FCGBP* as the most significantly upregulated gene associated with poor response to chemoradiotherapy.

To explore potential genetic determinants linked to the efficacy of neoadjuvant CRT, we conducted transcriptomic profiling using a published dataset (GSE35452) comprising rectal cancer tissue blocks (n = 46). Within this cohort, 22 patients (47.8%) were classified as nonresponders, while 24 patients (52.2%) were labeled as responders, forming the basis for the comparative analysis aimed at identifying predictive genetic biomarkers. Focusing on the cellular adhesion gene ontology (GO: 0007155), we pinpointed 6 probes covering 5 transcripts: *FCGBP*, *MUC4*, *DSG3*, *MUC5B*, and *SPON1*, all associated with CRT resistance (see Table 1 and Figure 1). Notably, among these 5 genes, *FCGBP* exhibited the highest mRNA expression level (log2 ratio = 1.5838, p = 0.0004). Subsequently, our investigation extended to a comprehensive examination of the expression levels and clinical significance of FCGBP in a larger rectal cancer cohort.

**Table 1:** Summary of differentially expressed genes associated with cell adhesion (GO:0007155) in relation to response to chemoradiotherapy in rectal carcinoma.*

**Probe**	**Comparison log ratio**	**Comparison *P*-value**	**Gene symbol**	**Gene name**	**Biological process**
203240_at	1.5838	0.0004	*FCGBP*	Fc fragment of IgG binding protein	binding of sperm to zona pellucida, cell adhesion
217109_at	1.2719	0.0006	*MUC4*	mucin 4; cell surface associated	cell adhesion, cell-matrix adhesion
235075_at	1.2535	0.0001	*DSG3*	desmoglein 3 (pemphigus vulgaris antigen)	cell adhesion, homophilic cell adhesion
213432_at	1.2364	0.0007	*MUC5B*	mucin 5B; oligomeric mucus/gel-forming	cell adhesion
209436_at	1.1393	<0.0001	*SPON1*	spondin 1; extracellular matrix protein	cell adhesion, multicellular organismal development
217110_s_at	1.0344	0.0047	*MUC4*	mucin 4; cell surface associated	cell adhesion, cell-matrix adhesion, transport

*Gene with log2 ratio >=1; comparison p-value <0.01 were selected.

### Clinicopathological features of the validation cohort in rectal cancer

We enrolled a cohort of 343 patients diagnosed with rectal adenocarcinoma who underwent neoadjuvant CRT, and their clinicopathological characteristics are summarized in Table 2. The majority of cases were male (n = 223, 65.0%), and most were below the age of seventy (n = 217, 63.3%). At the time of the initial clinical diagnosis, 210 patients (61.2%) presented with advanced tumoral status (cT3–T4), and 144 patients (42.0%) exhibited positive lymph node status (cN1–N2). Following CRT, 188 patients (54.8%) showed invasion depth beyond the muscularis propria (ypT3–T4), and positive lymph nodes (ypN1–N2) were observed in 118 patients (34.4%). Additionally, vascular invasion and perineural invasion were noted in 38 cases each (11.1%). To assess the efficacy of CRT in patients with rectal cancer, the tumor regression grade, as determined by the Dworak system, was employed [23]. Scores indicated that 73 cases (21.3%) exhibited little or no regression (grade 0–1), while 22 cases (6.4%) demonstrated complete regression (grade 4).

**Table 2:** Associations and comparisons between FCGBP expression and clinicopathological factors in 343 rectal cancer patients receiving chemoradiotherapy.

**Parameter**		**No.**	**FCGBP Expression**		**p-value**
			**Low Exp.**	**High Exp.**	
**Gender**	Male	223	119	104	0.622
	Female	120	62	58	
**Age**	<70	217	105	112	0.476
	≥70	126	66	60	
**Pre-Tx tumor status**	T1-T2	133	73	60	0.138
	T3-T4	210	98	112	
**Pre-Tx nodal status**	N0	199	115	84	0.001*
	N1-N2	144	56	88	
**Post-Tx tumor status**	T1-T2	155	105	50	<0.001*
	T3-T4	188	66	122	
**Post-Tx nodal status**	N0	225	131	94	<0.001*
	N1-N2	118	40	78	
**Vascular invasion**	Absent	305	162	143	0.001*
	Present	38	9	29	
**Perineurial invasion**	Absent	305	158	147	0.041*
	Present	38	13	25	
**Tumor regression grade**	Grade 0-1	73	22	51	<0.001*
	Grade 2~3	248	131	117	
	Grade 4	22	18	4	

Exp., expression; Tx, treatment; *, statistically significant.

### Immunointensity of FCGBP and its correlation with clinicopathological variables in rectal cancer

**Figure 2:**
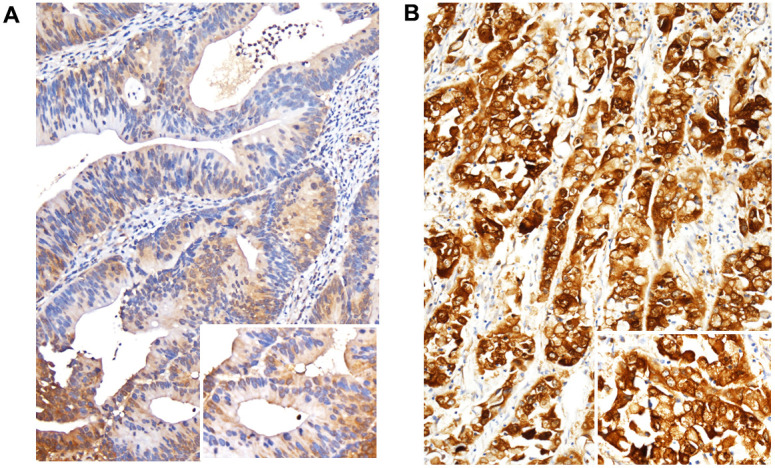
**Immunohistochemical expression of FCGBP.** Representative images of rectal cancer exhibiting FCGBP expression among tumor tissues. (**A**) Chemoradiotherapy responder with low FCGBP expression. (**B**) Chemoradiotherapy non-responder with high FCGBP expression. Representative images were captured at x200 magnification.

To assess the clinical significance of FCGBP in rectal cancer, we conducted immunohistochemical staining to evaluate its immunointensity and correlation with clinicopathological variables within our rectal cancer cohort. As illustrated in Figure 2A, 2B, the immunointensity of FCGBP staining exhibited a significant increase in tumor tissues associated with CRT-resistant rectal cancer. Comparative analysis with key clinical features outlined in Table 2 revealed a positive correlation between the immunointensity of FCGBP and lymph node metastases before CRT (p = 0.001), tumor invasion, and positive lymph node metastases after CRT (both p < 0.001), vascular invasion (p = 0.001), perineural invasion (p = 0.041), and a lower tumor regression grade (p < 0.001).

### The prognostic significance of FCGBP immunoexpression in rectal cancer

**Figure 3:**
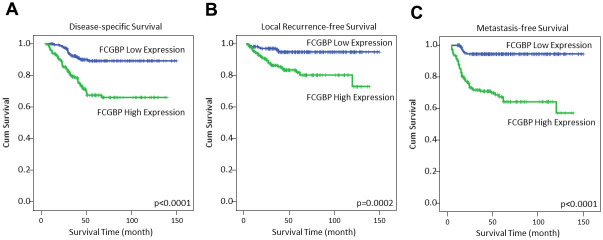
Kaplan–Meier analysis showed high expression of FCGBP was associated with inferior disease-specific survival (**A**), local recurrence-free survival (**B**) and metastasis-free survival (**C**).

As outlined in Table 3, rectal cancer was the cause of death for 63 patients (18%), with local recurrence and distant metastasis observed in 35 patients (10%) and 62 patients (18%), respectively. Subsequently, we conducted both univariate and multivariate analyses to assess the prognostic markers for disease-specific survival (DSS), local recurrence-free survival (LRFS), and metastasis-free survival (MeFS). At the univariate level, elevated FCGBP immunoexpression (Figure 3A–3C), low tumor regression grade, advanced post-CRT tumoral status, positive post-CRT lymph node status, and the presence of vascular invasion were significantly and adversely associated with all three endpoints (all p ≤ 0.0434). Furthermore, advanced pre-CRT tumoral status and the presence of perineural invasion were notably correlated with inferior DSS (p = 0.0074 and 0.018, respectively). In the multivariate analysis (Table 4), both high FCGBP immunoexpression and a low tumor regression grade independently emerged as unfavorable prognostic factors for all three endpoints (all p ≤ 0.025). Moreover, advanced pre-CRT tumoral status was also associated with inferior DSS (p = 0.036) in the multivariate analysis.

**Table 3:** Univariate log-rank analysis for important clinicopathological variables and FCGBP expression.

**Parameter**		**No. of case**	**DSS**		**LRFS**		**MeFS**	
			**No. of event**	**p-value**	**No. of event**	**p-value**	**No. of event**	**p-value**
**Gender**	Male	223	42	0.9755	9	0.2176	25	0.3460
	Female	120	21		26		37	
**Age**	<70	217	34	0.0728	23	0.9398	38	0.5771
	≥70	126	29		12		24	
**Pre-Tx tumor status**	T1-T2	133	15	0.0074*	13	0.6695	18	0.0663
	T3-T4	210	48		22		44	
**Pre-Tx nodal status**	N0	199	31	0.0932	19	0.4755	30	0.0697
	N1-N2	144	32		16		32	
**Post-Tx tumor status**	T1-T2	155	13	<0.0001*	8	0.0020*	15	<0.0001*
	T3-T4	188	50		27		47	
**Post-Tx nodal status**	N0	225	31	0.0022*	18	0.0434*	32	0.0077*
	N1-N2	118	32		17		30	
**Vascular invasion**	Absent	305	50	0.0023*	28	0.0379*	50	0.0136*
	Present	38	13		7		12	
**Perineurial invasion**	Absent	305	51	0.0180*	33	0.4263	53	0.1782
	Present	38	12		2		9	
**Tumor regression grade**	Grade 0-1	73	28	<0.0001*	14	0.0017*	25	<0.0001*
	Grade 2~3	248	34		21		36	
	Grade 4	22	1		0		1	
**FCGBP expression**	Low Exp.	171	16	<0.0001*	8	0.0002*	9	<0.0001*
	High Exp.	172	47		27		53	

DSS, disease-specific survival; LRFS, local recurrence-free survival; MeFS, metastasis-free survival; Exp., expression; Tx, treatment; *, statistically significant.

**Table 4:** Multivariate analysis.

**Parameter**	**DSS**			**LRFS**			**MeFS**		
	**HR**	**95% CI**	**p-Value**	**HR**	**95% CI**	**p-Value**	**HR**	**95% CI**	**p-Value**
**FCGBP high expression**	**2.391**	**1.280-4.203**	0.006*	**2.595**	**1.130-5.960**	0.025*	**5.373**	**2.582-11.183**	<0.001*
**Tumor regression grade**	**2.770**	**1.684-4.545**	<0.001*	**2.404**	**1.248 -4.651**	0.009*	**2.012**	**1.235-3.279**	0.005*
**Post-Tx tumor status**	1.910	0.989-3.689	0.054	2.020	0.867-4.704	0.103	1.557	0.828-2.927	0.169
**Pre-Tx tumor status**	**1.891**	**1.043-3.427**	0.036*	-	-	-	-	-	-
**Post-Tx nodal status**	1.133	0.653-1.967	0.657	1.262	0.611-2.608	0.529	1.120	0.640-1.962	0.691
**Vascular invasion**	1.421	0.727-2.776	0.304	1.687	0.681-4.181	0.259	1.178	0.591-2.347	0.642
**Perineurial invasion**	1.450	0.745-2.822	0.274	0.310	0.072-1.338	0.1116	1.076	0.513-2.257	0.846

DSS, disease-specific survival; LRFS, local recurrence-free survival; MeFS, metastasis-free survival; CI, confidence interval; HR, hazard ratio; Tx, treatment; *, statistically significant.

### Correlation of FCGBP with biological processes in rectal cancer

**Figure 4:**
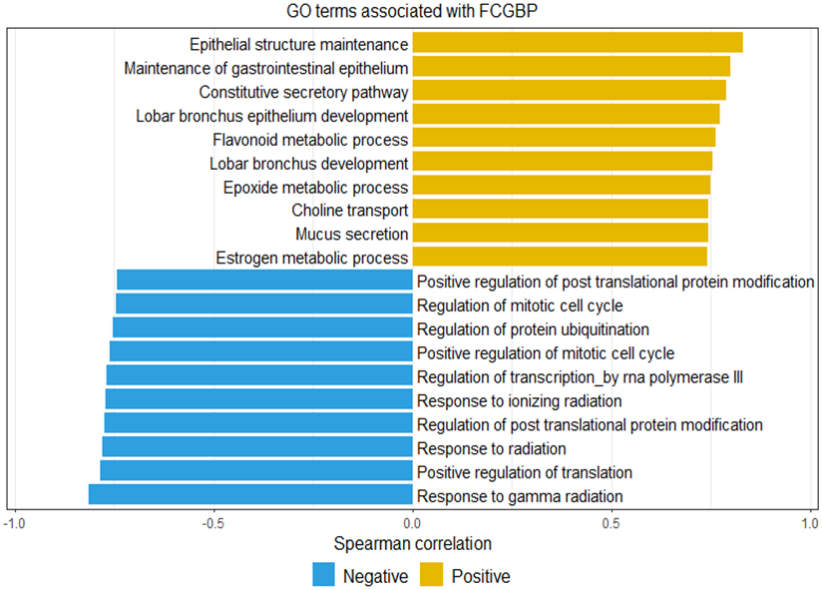
**Gene ontology terms representation based on the top 10 positive and negative correlations with *FCGBP* expression.** Yellow indicates positive correlations, and blue indicates negative correlations.

FCGBP’s impact on biological processes in rectal cancer was examined for both positive and negative correlations. A pathway enrichment analysis was performed to associate *FCGBP* expression with undisclosed biological functions in rectal cancer. Utilizing the same transcriptome database (GSE35452), we probed into the biological activities of FCGBP-interacting networks. The gene ontology (GO) terms, specifically those related to biological processes, were employed, and the pertinent biological functions of FCGBP in rectal cancer are presented in the Supplementary Table 1. Among these, epithelial structure maintenance and the flavonoid metabolic process, which is pertinent to radiation protection, were identified as significant processes positively correlated with *FCGBP* expression (illustrated in Figure 4). Notably, the term “response to gamma radiation” stood out as the most distinctive term negatively associated with *FCGBP* expression. The correlations between *FCGBP* expression and genes involved in these biological functions are detailed in Supplementary Table 2.

## DISCUSSION

In this investigation, we employed a transcriptome dataset, focusing our data mining efforts on the process of cell adhesion. Within this context, *FCGBP* emerged as the most markedly upregulated gene in CRT-resistant rectal cancer. Subsequent validation through immunohistochemical staining confirmed that heightened FCGBP expression is significantly correlated with an adverse response to CRT and unfavorable survival outcomes in our rectal cancer patient cohort. Consequently, the expression levels of FCGBP present a promising potential as a biomarker for predicting the prognosis of patients with rectal cancer undergoing preoperative CRT.

While the precise mechanisms underlying FCGBP expression in CRC development remain unclear, it is plausible to hypothesize that dysregulation of FCGBP may contribute to compromised mucosal immune defense, subsequently participating in intestinal inflammation. Given the pivotal role of chronic inflammation in tumor development, dysregulated FCGBP may be implicated in the carcinogenesis of CRC [24]. Similar to other mucins, FCGBP acts as a crucial mediator between an inflammatory milieu and a sustained physiological response, a process essential for cancer development and progression [25]. Chronic infections or injuries induce pro-inflammatory cytokines, leading to aberrant glycosylation and altered mucin expression [26]. These modifications in tumor-associated mucins furnish tumor cells with a diverse array of potential ligands capable of interacting with receptors on the cell surface throughout the course of tumor development and progression [27]. In a particular study, the suppression of MUC2, the primary component of intestinal mucus, resulted in the phosphorylation of STAT3 in tumor cells via the cytokine IL-6. This, in turn, stimulated the expression of IL-6 in both tumor and stromal cells, establishing an inflammatory microenvironment that promotes tumor growth [28]. IL-6, in this context, promotes epithelial-mesenchymal transition (EMT), a characteristic of tumor cells associated with invasion and metastasis [29]. In a separate study, FCGBP was found to be involved in EMT induced by the TGF-β1 pathway [14]. These collective findings suggest a potential significant role for FCGBP in the EMT process during tumor progression.

Extensive research has extensively documented the varied levels of FCGBP expression across diverse tumor types [14–19]. The observed disparities in FCGBP expression levels among different malignancies may signify distinct underlying functions and mechanisms. Tumors, in their quest to survive and proliferate in challenging environments, may utilize mucins to modulate the local microenvironment during inappropriate invasion and metastasis to various organ and tissue sites. Notably, the mucin layer could capture and retain biologically active molecules, such as growth factors, within the matrix, thereby facilitating tumor growth. Furthermore, mucins might serve as key players in aiding tumors to evade immune responses by creating an impenetrable barrier for immune effector cells or by inactivating immune effector cells through receptor–ligand interactions. This potential anti-immune response may indicate observed immune cell infiltrations in various malignancies, including glioma, hepatocellular carcinoma, and ovarian cancer [17–19]. Conversely, mucins may also exert a tumor-suppressive role [30]. For instance, the intestinal mucus layer can function as a physical barrier against dietary carcinogens or pro-carcinogenic microorganisms. Additionally, through interactions with membrane-bound mucins, secreted mucins might influence the differentiation and proliferation of epithelial cells—processes that are disrupted in cancerous growth. Given the emergence of immunotherapy as a promising anticancer treatment, investigations focusing on mucin-mediated tumor immunity are warranted. Understanding the intricate interplay between mucins and the immune system could provide valuable insights for developing novel therapeutic strategies in the realm of cancer treatment.

In Crohn’s disease and ulcerative colitis, the heightened expression of FCGBP may signify pathophysiologic changes in CRC development [20, 21]. However, numerous studies utilizing analytical approaches based on microarrays, online databases, and tissue microarray immunohistochemistry have revealed a significant diversity in FCGBP expression at both the mRNA and protein levels in CRC tissues compared with noncancerous colorectal tissues [31–33]. Some investigations have indicated that elevated FCGBP expression is associated with superior survival outcomes. It is noteworthy that none of these studies have explored the correlation between FCGBP expression levels and the treatment response to CRT in rectal cancer. In our current study, we made the novel observation that *FCGBP* mRNA expression is increased in rectal cancer tissues with CRT resistance through bioinformatic transcriptome analysis. This finding was further validated in our rectal cancer patient cohort, where elevated FCGBP expression at the protein level was linked to adverse pathological features following CRT, as well as shorter survival outcomes. Despite the retrospective nature of our rectal cancer cohort, the inclusion of a substantial number of tumor specimens in this study helps overcome this limitation.

High FCGBP expression has previously been correlated with chemotherapy resistance in advanced ovarian serous adenocarcinomas [19]. While the precise function of FCGBP in response to radiotherapy and/or chemotherapy remains incompletely understood, as these treatments induce DNA damage in cancer cells, FCGBP may contribute to tumor resistance against these DNA toxins by forming a molecular shield [30]. To deepen our understanding of the biological functions of FCGBP in rectal cancers, we evaluated the expression correlation between *FCGBP* and genes of interest involved in significant biological pathways identified from the enrichment analysis. We discovered that among the genes associated with epithelial structure maintenance (correlation coefficient > 0.5 and p < 0.05), *TFF1* (trefoil factor 1) and *TFF3* are significant oncogenic genes that promote CRC cell progression [34, 35]. Previous studies have shown that upregulation of *TFF1* and *TFF3* in cancer cells can facilitate EMT and confer resistance to chemotherapy and radiotherapy [36–39]. Notably, in addition to epithelial structure maintenance, our pathway enrichment analysis revealed a positive correlation between *FCGBP* expression and the flavonoid metabolic process. Flavonoids, a group of polyphenolic compounds, are recognized for their antioxidant properties and protective effects against radiation [40–42]. These findings suggest that FCGBP may be involved in radiation sensitivity through the metabolism of specific antioxidants. Furthermore, by examining genes involved in the flavonoid metabolic process, we found a positive correlation between *FCGBP* expression and *UGT1A1*, a crucial enzyme in the metabolism of chemotherapy drugs [43, 44]. The interaction between *FCGBP* expression and the genes regulating metabolism in cancer cells may contribute to CRT resistance. The observed negative correlation between the response to gamma radiation and *FCGBP* expression supports the notion that FCGBP modulates CRT sensitivity in rectal cancer. Further investigation into the correlation between FCGBP expression levels and the therapeutic efficacy of chemotherapy and radiotherapy holds the potential to yield novel insights into cancer treatment.

In conclusion, this study provides evidence indicating that heightened FCGBP immunoexpression is notably linked to a suboptimal response to CRT, as demonstrated through bioinformatic analyses of publicly available databases. The observed correlations between FCGBP expression, poor responses to CRT, and inferior survival outcomes were subsequently validated in our specific rectal cancer patient cohort. Consequently, the expression of FCGBP emerges as a potentially valuable predictive and prognostic marker for patients with rectal cancer undergoing CRT.

## MATERIALS AND METHODS

### Transcriptome profiling of rectal cancer

To investigate potential genes associated with the efficacy of neoadjuvant CRT, we utilized a published Gene Expression Omnibus dataset (GSE35452) comprising rectal cancer tissue blocks (n = 46) for transcriptomic profiling. In this dataset, biopsy specimens were collected during colonoscopic screening before CRT treatments. The raw microarray data (CEL files) obtained from the Human Genome U133 Plus 2.0 Array were processed using the statistical software Nexus Expression 3.0, and gene expression levels were determined using all probe sets without employing any filtering or mapping method. Based on the response to CRT, the samples were categorized into “nonresponders” and “responders” groups, and a supervised comparison between the two groups was conducted. We identified differentially expressed genes related to cell adhesion (GO: 0007155) and further refined the selection by focusing on genes with a p-value below 0.01 and a log2 ratio greater than 1 for subsequent analysis.

### Patient enrollment

The clinicopathological features and treatment outcomes were retrospectively reviewed and collected. Initial clinical staging was determined through colonoscopy, and patients without distant metastasis, as confirmed by chest X-ray and/or abdominopelvic computed tomography (CT), were included. Prior to proctectomy, all patients underwent a regimen of 45–50 Gy of radiotherapy in twenty-five fractions over five weeks, coupled with continuous infusional 5-fluorouracil-based treatment concurrently. For patients with tumoral status at least T3 or nodal status at least N1, additional chemotherapy (commonly using FOLFOX, CapOX, and 5-FU for at least 4 months) was administered before or after chemoradiotherapy. Sphincter-saving low anterior resection ensured a free circumferential resection margin for all patients. Lateral node dissection was performed when metastatic involvement was suspected, although none of our patients underwent lateral node dissection. Regular follow-up screenings occurred every 3–6 months for 5 years, involving a carcinoembryonic antigen blood test every 3 months, and an annual colonoscopy and abdominal CT or magnetic resonance imaging scan.

### Histopathological appraisal and immunohistochemical scoring

In the absence of patient clinical profiles, two experienced pathologists (Chien-Feng Li and Wan-Shan Li) meticulously examined all tumor samples to ensure a more objective assessment. The tumor and node stages before and after CRT were defined according to the seventh edition of the American Joint Committee on Cancer staging system. To assess the effectiveness of CRT in rectal cancer patients, we utilized the tumor regression grading system outlined by Dworak et al. [23]. Immunohistochemical staining procedures, consistent with protocols from our prior research [45], were employed. Tissue slides were incubated with the FCGBP primary antibody (Abcam; #Ab121202; Cambridge, UK). The immunointensity of FCGBP staining was categorized as follows: 0 (absent), 1+ (weak), 2+ (moderate), 3+ (strong). The H-score was applied to evaluate FCGBP immunoreactivity, calculated with the equation: H-score = ΣPi(i + 1), where Pi represents the percentage of stained tumor cells for each intensity (ranging from 0% to 100%), and i denotes the staining intensity (0 to 3+). The H-score, ranging from 100 to 400, was determined by combining the intensity and proportion of positively stained tumor cells. High FCGBP expression was defined as H-scores greater than or equal to the median of all scored instances.

### Gene ontology analysis

We employed gene set variation analysis (GSVA) to evaluate the enrichment levels of predefined gene sets in each sample [46], utilizing the R package GSVA. The biological process category from the Gene Ontology was obtained from the Molecular Signatures Database (MSigDB; https://www.gsea-msigdb.org/gsea/msigdb/). GSVA scores were calculated for each sample, offering an estimation of the enrichment levels of GO terms. To investigate GO terms correlated with FCGBP expression, we conducted a Spearman rank correlation test, with statistical significance determined by a Bonferroni-corrected adjusted p-value threshold of 0.05.

### Statistical analysis

All data underwent statistical analysis using Statistical Product and Service Solutions software version 22.0. The relationship between the expression of genes of interest, clinicopathological characteristics, and FCGBP expression levels was evaluated using Pearson’s chi-squared test. Survival curves were generated using the Kaplan–Meier method, and the log-rank test was applied to statistically compare two groups measured from surgery to the date of cancer death (DSS), first local recurrence (LRFS), or first metastasis (MeFS). Multivariate Cox proportional hazards regression analysis, incorporating parameters with prognostic utility identified at the univariate level, was employed to identify independent prognostic variables. Statistical significance was determined using a two-tailed test, with a p-value below 0.05 considered significant.

### Availability of data

Upon reasonable requests, the datasets generated and analyzed in the course of the present study are available from the corresponding author.

## Supplementary Material

Supplementary Table 1

Supplementary Table 2
